# Supplementary material for: Resolving the full spectrum of human genome variation using Linked-Reads
Source: Genome Res. 2019 Apr;29(4):635–45. doi: 10.1101/gr.234443.118 (PMC6442396; doi:10.1101/gr.234443.118)
Supplement: Supplemental Material [file supp_gr.234443.118_Supplemental_longranger-2.2.zip › longranger-2.2.tar.bz2!/lr2.2-public/lib/stan/cmdstan/stan/doc/api/html/inverse__softmax_8hpp_source.html]

Stan: src/stan/math/functions/inverse\_softmax.hpp Source File


|  |  |
| --- | --- |
|  | Stan  2.5.0  probability, sampling & optimization |


- Main Page
- Related Pages
- Namespaces
- Classes
- Files

- File List
- File Members

All Classes Namespaces Files Functions Variables Typedefs Enumerations Enumerator Friends Macros Pages

- src
- stan
- math
- functions

inverse\_softmax.hpp

Go to the documentation of this file.

1 #ifndef STAN\_\_MATH\_\_FUNCTIONS\_\_INVERSE\_SOFTMAX\_HPP

2 #define STAN\_\_MATH\_\_FUNCTIONS\_\_INVERSE\_SOFTMAX\_HPP

3

4 #include <boost/math/tools/promotion.hpp>

5 #include <stdexcept>

6 #include <boost/throw\_exception.hpp>

7

8 namespace stan {

9  namespace math {

10

33  template <typename Vector>

34  void inverse\_softmax(const Vector& simplex, Vector& y) {

35  using std::log;

36  if(simplex.size() != y.size())

37  BOOST\_THROW\_EXCEPTION(std::invalid\_argument ("simplex.size() != y.size()"));

38  for (size\_t i = 0; i < simplex.size(); ++i)

39  y[i] = log(simplex[i]);

40  }

41

42  }

43 }

44

45 #endif

stan::math::inverse\_softmax

void inverse\_softmax(const Vector &simplex, Vector &y)

Writes the inverse softmax of the simplex argument into the second argument.

**Definition:** inverse\_softmax.hpp:34

stan::math::log

Eigen::Matrix< T, Rows, Cols > log(const Eigen::Matrix< T, Rows, Cols > &m)

Return the element-wise logarithm of the matrix or vector.

**Definition:** log.hpp:16

stan::agrad::log

fvar< T > log(const fvar< T > &x)

**Definition:** log.hpp:15


---

[ Stan Home Page ]

*© 2011–2014,
Stan Development Team.*
